# Inhomogeneities in PNIPAM Aqueous Solutions: The Inside View by Spin Probe EPR Spectroscopy

**DOI:** 10.3390/polym13213829

**Published:** 2021-11-05

**Authors:** Ekaterina M. Zubanova, Sergei V. Kostjuk, Peter S. Timashev, Yury A. Rochev, Alexander I. Kokorin, Mikhail Ya. Melnikov, Elena N. Golubeva

**Affiliations:** 1 Faculty of Chemistry, Lomonosov Moscow State University, 1-3, Leninskie gory, 119991 Moscow, Russia; timashev.peter@gmail.com (P.S.T.); melnikov46@mail.ru (M.Y.M.); legol@mail.ru (E.N.G.); 2 Research Institute for Physical Chemical Problems of the Belarusian State University, 14, Leningradskaya str., 220030 Minsk, Belarus; kostjuks@bsu.by; 3 Institute for Regenerative Medicine, Sechenov First Moscow State Medical University, 8-2, Trubetskaya str., 119992 Moscow, Russia; yury.rochev@nuigalway.ie; 4 Faculty of Chemistry, Belarusian State University, 220006 Minsk, Belarus; 5 N.N. Semenov Federal Research Center for Chemical Physics, Russian Academy of Sciences, Kosygin st. 4, 119991 Moscow, Russia; alex-kokorin@yandex.ru; 6 National University of Ireland Galway, University Road, H91 TK33 Galway, Ireland; 7 Plekhanov Russian University of Economics, Stremyannyi per., 117997 Moscow, Russia

**Keywords:** thermoresponsive polymers, electronic paramagnetic resonance, spin probe, nitroxides, coil to globule

## Abstract

Coil to globule transition in poly(N-isopropylacrylamide) aqueous solutions was studied using spin probe continuous-wave electronic paramagnetic resonance (CW EPR) spectroscopy with an amphiphilic TEMPO radical as a guest molecule. Using Cu(II) ions as the “quencher” for fast-moving radicals in the liquid phase allowed obtaining the individual spectra of TEMPO radicals in polymer globule and observing inhomogeneities in solutions before globule collapsing. EPR spectra simulations confirm the formation of molten globules at the first step with further collapsing and water molecules coming out of the globule, making it denser.

## 1. Introduction

Thermoresponsive polymers are of great interest because they undergo coil-to-globule transitions of single-polymer chains in polar solvents near the lower critical solution temperature (LCST) [1,2,3]. This peculiarity gives them potential for biomedical and pharmaceutical applications such as drug and gene delivery, tissue engineering, cell expansion, sensors, microarrays, and imaging [4,5,6,7,8,9]. Phase transition in the thermoresponsive polymers solutions passes due to molecular interaction and the cohesion of solvent molecules with hydrophilic fragments in the polymer chains [10,11]. Hydrophilic groups form hydrogen bonds with water molecules at low temperatures, resulting in good solubility in aqueous solutions. Increasing the temperature leads to the degradation of the hydrogen bonds system and the formation of an intramolecular interaction between polymers chains, further collapsing the polymer globule. Poly(N-isopropylacrylamide) (PNIPAM) is one of the most studied thermoresponsive polymers, which has LCST in a physiologically relevant temperature range of ≈32 °C in aqueous solutions [1]. Macroscopic methods (turbidimetry, DSC, etc.) usually fix sharp and reversible changes of PNIPAM properties in the vicinity of LCST [11,12]. In addition, the formation of small, even nanoscopic inhomogeneities of polymer gels or films in different solvents before LCST is proved by continuous-wave electron paramagnetic resonance spectroscopy (CW EPR) [13,14,15]. The EPR spectra of paramagnetic molecules (spin probes) are sensitive to the microenvironment and can give valuable information concerning collapse processes at the molecular level [14,16,17]. Stable nitroxide radicals containing a >N–O●·group have been the most popular spin probes during the last 55 years [17,18]. Hyperfine interaction of the unpaired electron spin with the magnetic moment of 14N (S = 1) nucleus leads to splitting of the EPR signal of nitroxides to three lines. Local polarity, viscosity, and the ability of a media to form hydrogen bonds influence the electron density distribution in the spin probe, affecting the shape of the EPR signal and spin-Hamiltonian parameters (g-tensor and hyperfine splitting (hfs) tensor), which can be estimated by modeling the EPR spectra. The small amphiphilic radical 2,2,6,6-tetramethylpiperidin-1-yl)oxyl (TEMPO) (ca. 6.7 Å in diameter) is a powerful tool to detect and control the formation of polymer globules [14,19,20,21]. It is known that the micropolarity in polymeric globules of thermoresponsive polymers is significantly lower than the polarity of water and is close to that of chloroform [18]. A lower polarity and higher viscosity of the globules [15] result in changes of spin Hamiltonian parameters and the line widths of TEMPO spectra compared with those in aqueous solution, reflecting a decrease of the amplitudes of the spectral lines belonging to the radicals in the solution and the appearance of broader components of the TEMPO spectrum corresponding to probes localized in the globules. This effect was used by Junk et al. [19] to reveal the formation of heterogeneities in thin photocrosslinked films of PNIPAM notably earlier and later the LCST detected by macroscopic methods. However, the spin probe technique was not applied to study coil-to-globule transitions and the nature of inhomogeneities in PNIPAM aqueous solutions up to now. In the present paper, we applied CW EPR to study the structure and features of formation of nano- and/or micro heterogeneities of PNIPAM with two different polydispersities and the dynamics of spin probes inside the globules and in aqueous solutions upon heating from room temperature.

## 2. Materials and Methods

### 2.1. Substances

The stable radical (2,2,6,6-tetramethylpiperidin-1-yl)oxyl (TEMPO) and copper(II) chloride dihydrate CuCl_2_·2H_2_O purchased from Sigma-Aldrich were used without further purification. N-Isopropylacrylamide (NIPAM) (99%, Acros, Geel, Belgium) was recrystallized from solution in *n*-hexane, dried in vacuum, and then stored under argon atmosphere. 2,2′-Azobis(2-methylpropionitrile) (AIBN) (98%, Sigma-Aldrich, Burlington, MA, USA) was recrystallized from ethanol and dried in vacuum at 20 °C. Benzene (anhydrous, 99.8%, Sigma-Aldrich, USA) and *n*-hexane (reagent grade, Ekos-1) were used as received.

### 2.2. Polymer Synthesis

Two poly(N-isopropylacrylamide) (PNIPAM) samples I and II were synthesized via conventional radical and RAFT polymerization, respectively. The sample I (number-average molar mass M_n_ = 175.5 kDa, polydispersity index Đ = 4.3; yield: 97%) was prepared by free radical polymerization in benzene at 60 °C for 24 h using azobisisobutyronitrile (AIBN) as an initiator according to the procedure reported in [20]. Then, the reaction mixture was precipitated in n-hexane. Then, the obtained polymer was purified by dissolving in acetone followed by precipitation in n-hexane at least three times, and the product was dried at 45 °C in a vacuum oven. Sample II (M_n_ = 107.6 kDa, Đ = 2.05; yield: 95%) was prepared by RAFT-mediated radical polymerization. The polymerization was carried out in benzene at 60 °C for 24 h using 2-(dodecylthiocarbonothioylthio)-2-methylpropionic acid as the RAFT agent. The polymerization was conducted in an argon atmosphere in a Schlenk reactor equipped with a magnetic stir bar. The reactor was charged by N-isopropylacrylamide (0.502 g, 4.44 mmol), vacuumed, and filled with argon. Then, 0.22 mL of 0.02 M benzene solution of RAF-agent, 1.11 mM of 0.02 M benzene solution of AIBN, and 2.87 mL of benzene were added into the reactor. The mixture was bubbled with argon for 30 min, and then, the reactor was placed into an oil bath heated to 60 °C. After 24 h, the reactor was opened and frozen with liquid nitrogen. The polymer purification was performed similarly to the purification of sample I (see above).

### 2.3. Polymers Characterization

#### 2.3.1. H NMR Spectroscopy

**Figure A1:**
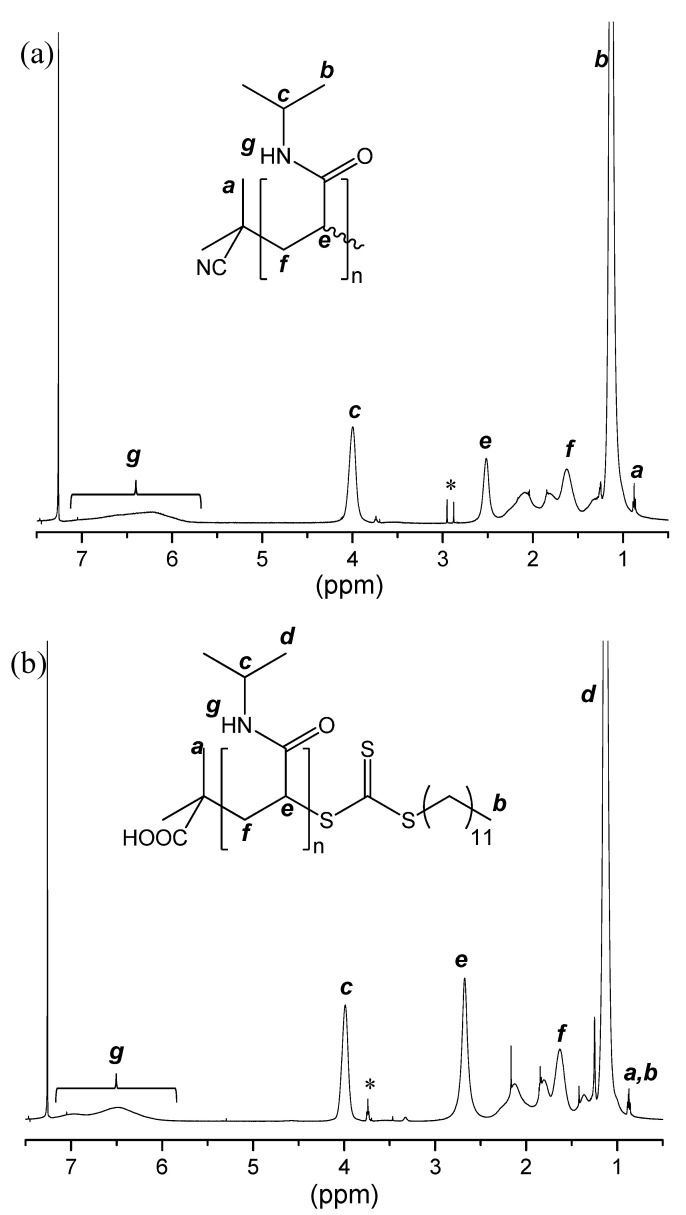
^1^H NMR spectra of (**a**) sample I and (**b**) sample II. The traces of solvents are marked by asterisks.

^1^H NMR (500 MHz) spectra were recorded in CDCl_3_ at 25 °C on a Bruker AC-500 (Bruker, Karlsruhe, Germany) spectrometer calibrated relative to the residual solvent resonance. The ^1^H NMR spectra of sample I and sample II are presented in Figure A1 (see Appendix A).

#### 2.3.2. Spectrophotometry

The temperature transition was monitored by UV-Vis spectrophotometry using a Victor Nivo instrument (Perkin Elmer, Waltham, MA, USA) at a wavelength of 405 nm. The studied polymer solution was poured into a 96 (48)-well plate, and the absorbance at each point was registered. The range of measurement was 4–40 °C. The cloud points were determined at 10% of the transmission reduction.

#### 2.3.3. Differential Scanning Calorimetry (DSC)

The DSC studies were carried out using a NETZSCH STA 449 F3 synchronous thermal analyzer (Selb, Germany) in a helium atmosphere at gas flow rates of 70 mL/min (main) and 50 mL/min (protective). An aluminum crucible was used with a solution weight of 30–45 mg. The calibration of the temperature and sensitivity of the device was carried out using standard samples of adamantane, indium, and distilled water. The aqueous solution of the polymer sample (5 wt %) was heated and cooled at 2 K/min. TGA measurements were performed with the same device using solid PNIPAM samples at heating rate of 20 °C min^−1^ under nitrogen flow.

#### 2.3.4. Size Exclusion Chromatography

Size exclusion chromatography was performed using an Ultimate 3000 Thermo Scientific chromatographic complex (Thermo Fisher Scientific, Waltham, MA, USA) equipped with PLgel precolumn guard (Agilent, Santa Clara, CA, USA, size 7.5 × 50 mm, particle size 5 μm) and PLgel MIXED-C column (Agilent, size 7.5 × 300 mm, particle size 5 μm) thermostated at 50 °C. The elution was performed in the isocratic mode with DMF containing 0.10 M LiBr at a flow rate of 1.0 mL min^−1^. SEC traces were recorded as mentioned above, and polymethylmethacrylate standards (ReadyCal Kit, PSS GmbH) with Mw/Mn ≤ 1.05 were used to calculate Mw/Mn.

### 2.4. EPR Samples Preparation

In all cases, ≈0.5 mM TEMPO and 10 wt % PNIPAM freshwater solutions were prepared, dissolving the required predefined amounts of TEMPO radical and PNIPAM polymer. In the first step, the dissolution of TEMPO was performed at room temperature using ultrasonication. Then, PNIPAM was added, and the mixture was aged at 4 °C for 24 h until the complete dissolution of the polymer. The solutions were put into glass tubes with 2 mm inner diameter; then, the tubes were sealed to prevent water evaporation.

### 2.5. EPR Measurements

EPR spectra were recorded using X-band spectrometer Bruker EMX-500 (Bruker, Karlsruhe, Germany). The temperature of the samples was varied in the range of 300–353 K using the flow of nitrogen gas. The thermostatic device from Bruker was used; the accuracy of the temperature setting was about ±0.5 K. Each sample was left at the particular temperature for precisely 5 min for equilibration before recording. At 305 K, the waiting time was 60 min to equilibrate the samples. Typical parameters of the spectra recording were a microwave power of 0.8 mW, modulation amplitude of 0.04 mT, and a sweep width of 8 mT. «Quenching» of fast spin probes in water was performed by adding 10 mg of CuCl_2_∙2H_2_O to 0.5 mL PNIPAM solution, as recommended in [21].

### 2.6. Data Analysis

The integration of EPR spectra and the amplitude measuring were performed by the EsrD program developed at the Chemistry Department of Lomonosov Moscow State University [16]. For LCST measurements, the amplitude value of TEMPO EPR spectra was normalized to the Q-factor [22] at each temperature and to the amplitude of the left component of the spectra at 300 K. All spectra simulations were performed using homemade scripts for the MATLAB program employing an Easyspin (v. 5.2.28) toolbox [23]. Spectra of TEMPO radical in solutions (type ***A***) were simulated using a model based on fast motion implemented as a ‘garlic’ function in Easyspin. Simulations of the spectra of radicals in polymer globule (type ***B***) were made in a slow-motion regime using a ‘chili’ function from the Easyspin program. The slow-motion model ‘chili’ is based on the Schneider–Freed theory [24], solving equations for slow tumbling nitroxides. Anisotropic values of Spin-Hamiltonian parameters (*g*-tensor and hyperfine splitting (hfs) tensor, usually denoted as *a*-tensor) were averaged to obtain *g*_iso_ and *a*_iso_ values, the rotational correlation time tensor (*τ_corr_*), denoted as the average time required for the rotation of a molecule at one radian, was calculated from averaged rotational diffusion constant. Fitting errors for *g*_iso_ and *a*_siso_ were ±0.00003 and ±0.01 mT, respectively. More details of the spectra simulation are presented in Appendix A.

## 3. Results

**Figure A2:**
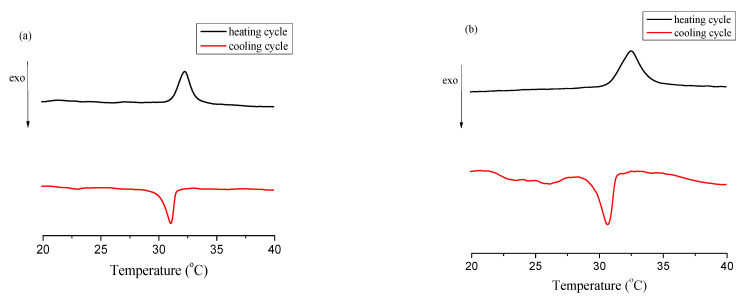
DSC thermograms of 5 wt % aqueous solutions of poly(N-isopropylacrylamide): (**a**) sample I and (**b**) sample II.

**Figure A3:**
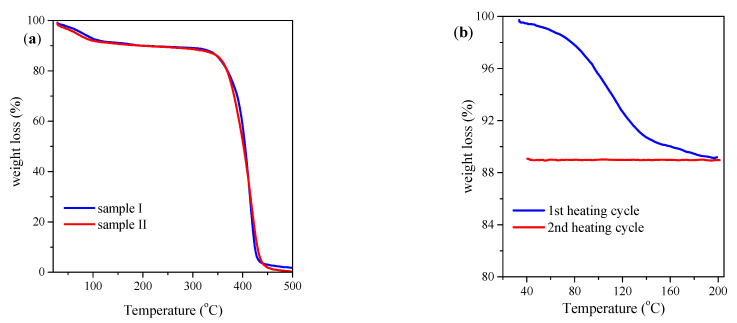
TGA curves of (**a**) sample I and sample II studies in this work and (**b**) sample II before and after annealing at 470 R for 15 min.

According to DSC (Figure A2 in Appendix A) data from the heating step, the measured LCST of the polymers I and II in aqueous solutions was ≈305 K and did not depend on the synthesis method and molar mass distribution of polymers, which is typical for PNIPAM solutions [25]. TGA measurements showed that both PNIPAM samples are stable at the temperatures used in this work (≤353 K), and the thermal decomposition (5 wt % weight loss) began at ≈625 K (Figure A3a). Note that the weight loss between 320 and 400 K (Figure A3a) is consistent with the presence of ca. 10–12 wt % of water in poly(N-isopropylacrylamide) samples. This is confirmed by the disappearance of this weight loss after annealing the samples at 470 K for 15 min (Figure A3b).

**Figure 1:**
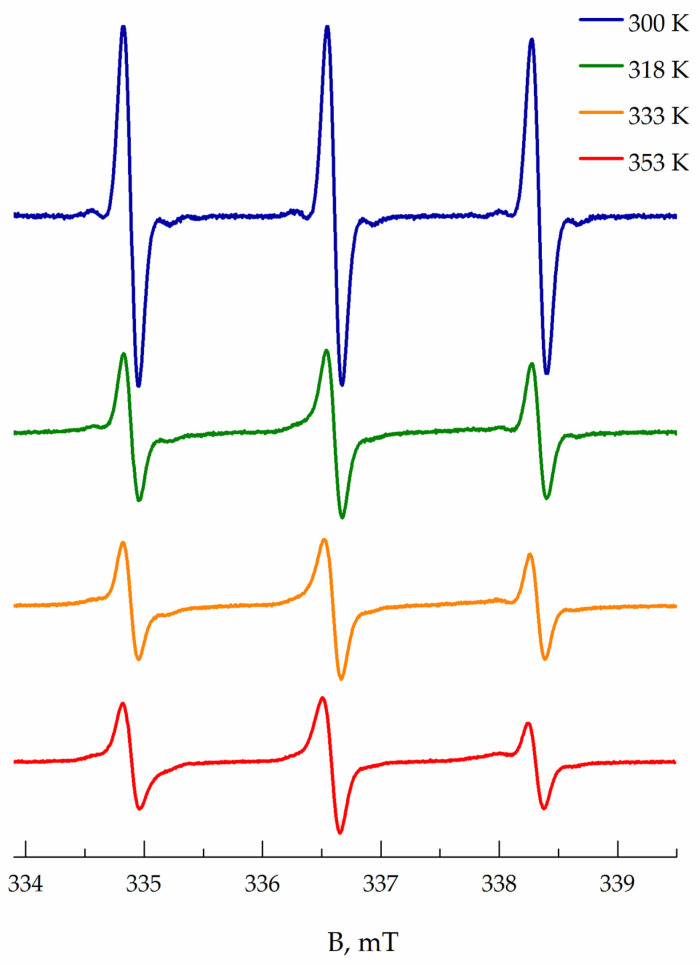
Normalized EPR spectra of TEMPO radical in 10 wt% PNIPAM aqueous solutions at 300–353 K registered upon heating.

**Figure A4:**
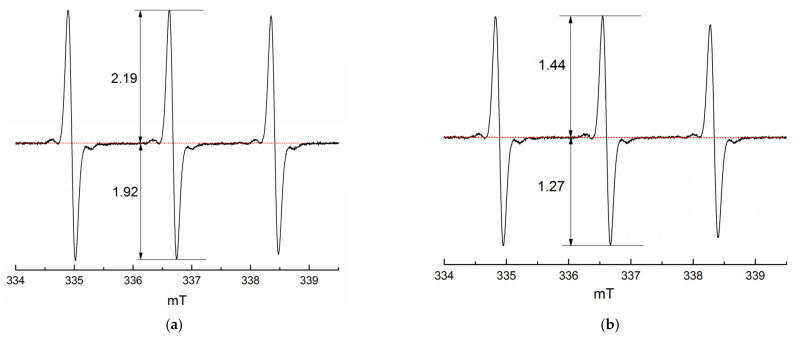
EPR spectra of TEMPO radical in water (0.3 mM) (**a**) and 10% PNIPAM solution (0.5 mM) (**b**) at 298 K. Arrows show amplitude value of spectra above and below the baseline. Red dot line shows baseline.

At temperatures below LSCT (305 K), the EPR spectrum of TEMPO in PNIPAM solution (sample I, 10 wt%) and TEMPO spectrum in water have a shape close to the isotropic fast-motion limit (Figure 1). The EPR spectrum of TEMPO in the polymer II solution and its changes during heating were very similar to those of polymer I. However, all these spectra (see Figure A4 in Appendix A) are slightly asymmetric; i.e., the signal amplitude above the baseline is bigger than that below it. This asymmetry may appear due to the following reasons. Firstly, the existence of the solvent shells with different polarities around TEMPO molecules in aqueous media revealed by the analysis of *Q*-band EPR spectra was postulated by Hunold et al. [26]. This fact leads to a bimodal distribution of magnetic and dynamic parameters of TEMPO probes, which manifest itself in asymmetric signals due to the superposition of the spectra. Secondly, the asymmetry of the nitroxide signal can be caused either by the intermolecular spin-exchange interaction between spin probes in solutions [27,28] or by mixing with the dispersion signal of the resonance effect [29]. Fortunately, in our case, the observed effects are too small and can be neglected to simplify the spectra modeling in aqueous solutions. According to our simulation, the TEMPO spectrum in water at room temperature corresponds to probes with a *g*_iso_ value equal to 2.00579 and the hfs constant *a*_iso_ equal to 1.73 mT. The isotropic rotational correlation time (*τ_corr_*) is about 11 ps. TEMPO in 10 wt % PNIPAM solution has parameters typical for less polar media: *g*_iso_ equal to 2.00585 and *a*_iso_ is equal to 1.72 mT, wherein *τ_corr_* = 16 ps, manifesting the higher viscosity of polymer solution. A simple estimation of viscosity using the Stocks–Einstein equation from rotational correlation time shows increasing viscosity of 10 wt% PNIPAM solution below LCST in ≈1.5 times compared to pure water.

**Figure 2:**
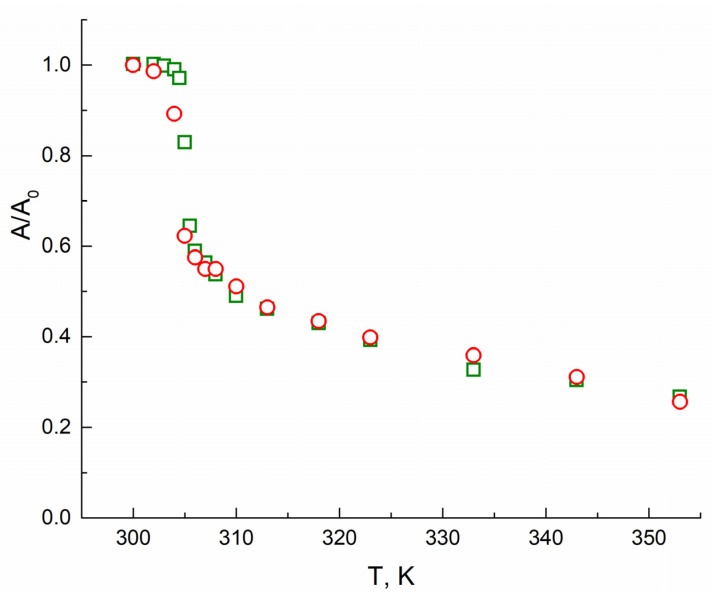
The normalized amplitude of the high-field component of TEMPO EPR signal in PNIPAM solution (green squares correspond to polymer I, red circles correspond to polymer II) vs. temperature.

At temperatures higher than 305 K, the intensity of the TEMPO spectra decreases rapidly, and the lines shape changes: the spectra become broader, and the central line turns to more asymmetric. At 320 K and higher, the components in the high-field region corresponding to the probe in the hydrophobic globule clearly manifest themselves in the spectra. Changes in the lines’ shape can be empirically presented as the amplitude A of the high field component vs. temperature T (Figure 2). As seen from Figure 2, this dependence allows measuring the LCST of polymer solutions. A fast drop of the amplitude occurs due to the appearing of the more broadened and hence less intensive signal of TEMPO radicals located in globules.

At temperatures above the LCST, the TEMPO spectrum in the PNIPAM solution appears to be the sum of at least two signals. One of them belongs to the radicals in the solution (type ***A***). At the same time, the position and shape of the second signal (denoted as the spectrum of ***B***-type probes) manifest a more hydrophobic local environment and a hindered rotation of TEMPO radicals comparing to ***A*** probes. The simulation of experimental spectra as the sum of the spectra of two probes applies a considerable number of parameters, the variation of which leads to similar changes in EPR spectra. For example, a shift of the components can occur both due to changes in the hfs constant or rotational correlation time. The use of the spin-Hamiltonian and dynamic parameters of radicals ***A*** at different temperatures determined from the temperature dependence of TEMPO spectra in an aqueous solution makes it possible to slightly reduce the number of parameters to be varied in the modeling. Nevertheless, the parameters of probes ***B*** cannot be quantitatively determined from the simulation of experimental spectra due to their simultaneous influence on the shape of the spectral line and overlapping signals of probes ***B*** with the component corresponding to the hyperfine splitting on ^13^C nuclei of TEMPO, the so-called satellite in the probes ***A*** spectrum. The spectrum of ***B*** probes can be elucidated by subtracting the spectrum of centers ***A*** from the total spectrum or as the spectra of polymer films swollen in the probe solution [19]. Nevertheless, the subtracting does not allow obtaining a fully “pure” single-particle spectrum and may lead to artifacts in the line shape.

**Figure A5:**
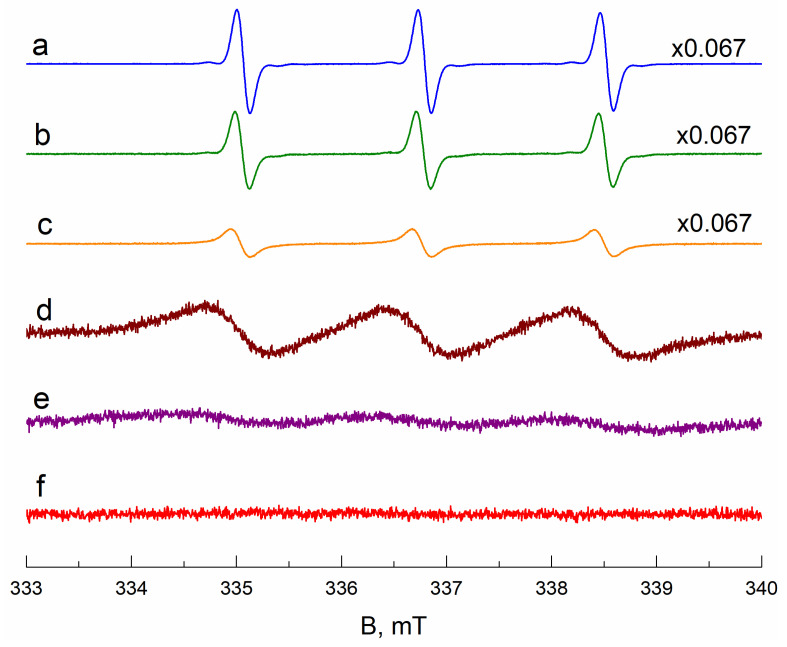
EPR spectra of TEMPO radical in water in the presence of Cu(II) ions: (**a**)—[Cu(II)] = 0, [TEMPO] = 0.5 mM; (**b**)—[Cu(II) = 0.003 M, [TEMPO] = 0.33 mM; (**c**)—[Cu(II)] = 0.01 M, [TEMPO] = 0.25 mM; (**d**)—[Cu(II)] = 0.1 M, [TEMPO] = 0.5 mM; (**e**)—[Cu(II)] = 0.2 M, [TEMPO] = 0.5 mM; (**f**)—[Cu(II)] = 0.3 M, [TEMPO] = 0.5 mM. Spectra (**a**–**c**) were divided by 15 for better representation and comparison with (**d**–**f**).

The perspective method to get the individual spectrum of radicals inside a polymer globule is “quenching” the signal of rapidly rotating radicals ***A*** due to the spin-exchange broadening of EPR lines [21]. A Heisenberg exchange interaction occurs between unpaired electrons as a result of the collision of two paramagnetic particles, leading to an exchange of spin states between partners. In turn, the exchange of spin states between partners leads to a change in the width of the EPR line of the paramagnetic particle up to its confluence with the baseline. Such broadening may appear in the presence of some inert paramagnetic particles, e.g., Cu(II) ions, not chemically interacting with components of solutions. Figure A5 presents the change of the EPR spectra of TEMPO radicals in aqueous solutions in the presence of Cu(II) ions. Adding small amounts of the paramagnetic ions firstly leads to minor broadening of the signal of motile radicals in the solution. Further increasing of Cu(II) concentration continues to broaden the TEMPO signal up to the baseline. The optimal concentration of Cu(II) ions is about 0.2 M, and this amount was used for “quenching” the signal of radicals ***A*** in PNIPAM (polymer I) solutions.

**Figure 3:**
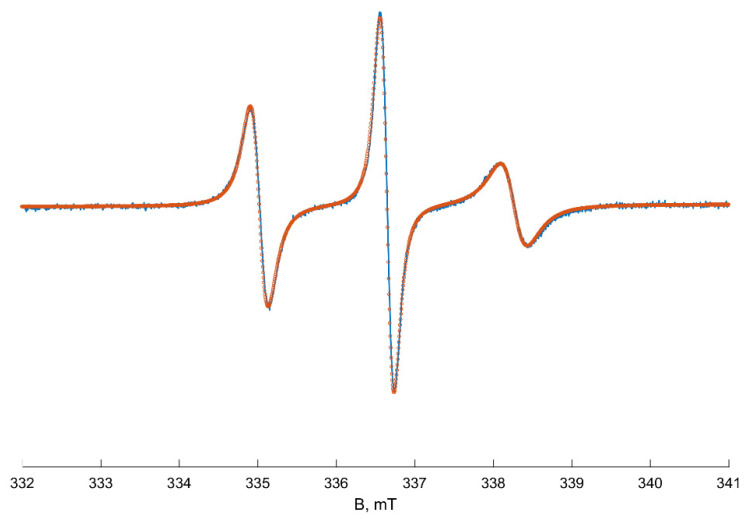
EPR spectrum of TEMPO radical in PNIPAM solution at 353 K in the presence of Cu(II) ions. Blue line—experimental spectrum, red dots—simulated spectrum.

**Figure A6:**
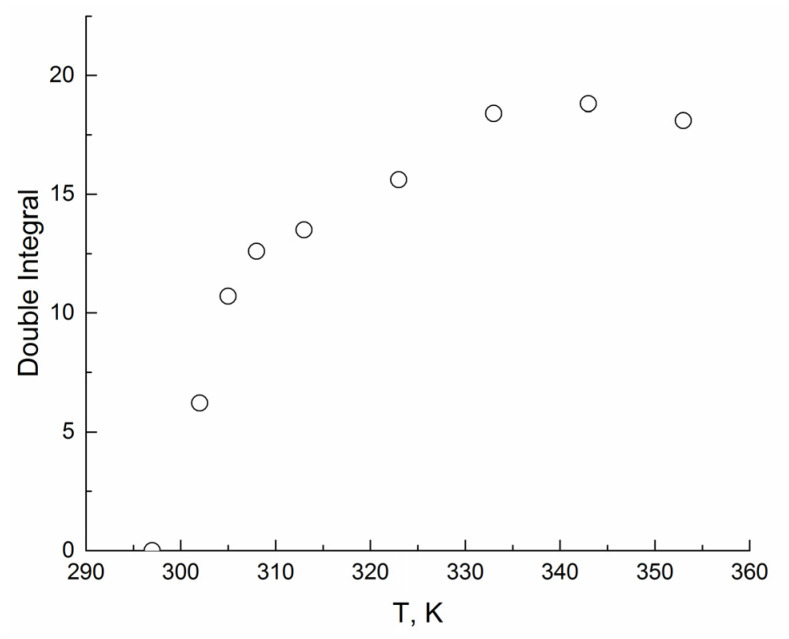
Change of double integral of EPR spectra of TEMPO in PNIPAM aqueous solution in the presence of Cu(II) vs. temperature.

Due to the spin exchange between Cu(II) ions and TEMPO, the experimental spectrum transfers practically to a base line at the temperature range of 295–301 K. At 302 K, a new slight signal of slow-moving radicals appears, and its intensity and double integral increase with temperature rising (see Figure A6, Appendix A). We suppose that the new signal corresponds to TEMPO radicals located in inhomogeneities of the polymer solution and not contacted with the quencher. Over 305 K, polymer globules start collapsing, and the spectra become more intensive due to an increase in the fraction of the radicals captured by polymer globules. Heating up to 323 K leads to a further increase in the TEMPO signal, but the lines’ shape does not change noticeably. At 333–353 K, the components of the spectra narrow, apparently, because of the increasing mobility of TEMPO radicals. The number of spin probes that depends linearly on the double integral of EPR spectra stays constant at 333–353 K. We assume that the spectrum at 353 K shown in Figure 3 matches the TEMPO spectra of the radicals in dense PNIPAM globules (probes ***B***). According to our simulation results, these spectra correspond to the anisotropic rotation of TEMPO radicals with *g*_iso_ and *a*_iso_ equal to 2.00615 and 1.60 mT, respectively. Such value of the isotropic hfs constant matches to the local polarity close to that of the chloroform or low-molecular alcohols [30]. Rotational movements of radicals are anisotropic and more hindered along the *x*-axis: *D*_x_ = 7.6 × 10^6^ s^−1^ < *D*_z_ = 8.7 × 10^7^ s^−1^ < *D*_y_ = 5.9 × 10^8^ s^−1^, where *D*_x,y,z_ are the components of the rotational diffusion tensor. The obtained magnetic and dynamic parameters of TEMPO radicals in PNIPAM globules (probes B) were used in our simulations of the total spectra series (Figure 1).

**Figure A7:**
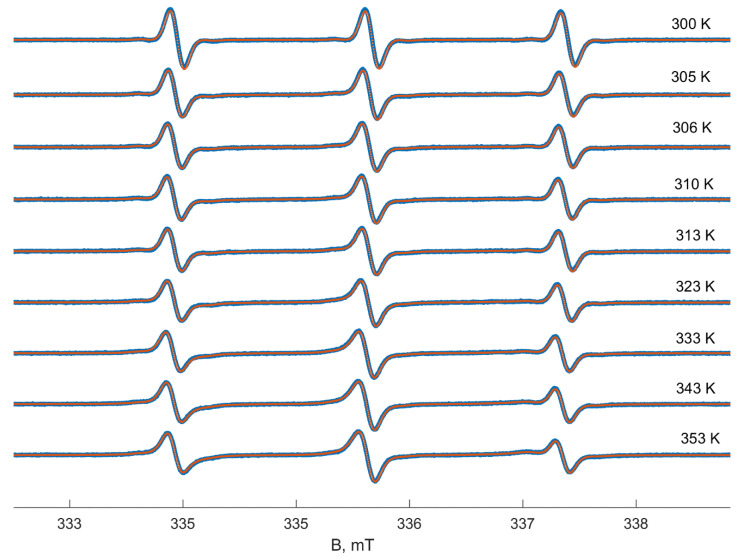
Simulation of spectra of system TEMPO/PNIPAM/H_2_O at different temperatures. Blue lines—experimental spectra, red circles—simulation spectra.

All spectra of the samples I and II at temperatures above 305 K were excellently fitted as a sum of the spectra of two types: “hydrophilic, fast” ***A*** probes and “hydrophobic, slow” ***B*** probes (Appendix A, Figure A7). The root-mean-square deviation obtained from simulations of all fitted spectra was less than 1%. The complete simulation results obtained from EPR spectra fitting at several temperatures are collected in Appendix A in Table A1. The difference between the two samples comes out only as the content of the ***B*** probes at 305 K: 26% in sample I and 44% in sample II. This difference may occur due to various polydispersities of polymers I (*Đ* = 4.3) and II (*Đ* = 2.05). Actually, the content of macromolecules with similar molecular mass is bigger in polymer II, leading to a more rapid globule collapse and the capturing of bigger content of the TEMPO radical. The simulation results of the EPR spectra of samples I and II registered higher than 305 K are similar, so only the simulation results obtained for sample II will be discussed further.

**Figure 4:**
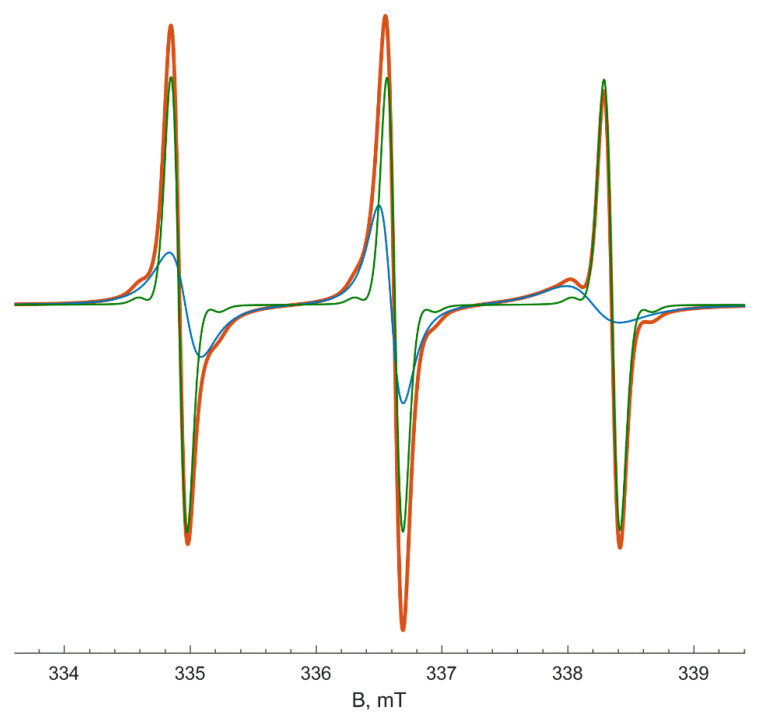
Decomposition of fitted EPR spectrum (red) of TEMPO radical in 10 wt % PNIPAM solution at 333 K into two signals: type ***A*** radicals (green) and type ***B*** radicals (blue).

Figure 4 illustrates the fitted EPR spectrum recorded at 333 K as a superposition of two individual spectra. The spectrum of probes A looks like a “fast limit” spectrum of TEMPO radicals dissolved in water. Its EPR parameters become less polar with heating due to decreasing the polarity of liquids (for example, the dielectric constant ε of water changes from 78 to 60 at temperatures 298 and 350 K, respectively) [31]. A hyperfine coupling constant also decreases from 1.73 to 1.71 mT, and giso increases from 2.00588 to 2.00598. The rotation correlation time of A probes decreases with heating from 10 to 1 ps due to the diminishing water viscosity [32]. The magnetic and dynamic parameters of type A probes after LCST do not depend on PNIPAM concentration and are similar to those for the pure aqueous solution without PNIPAM.

The spectrum of ***B*** probes (radicals located in hydrophobic globules) is more broadened and looks like a «slow-motion» signal. The EPR spectrum of type ***B*** at 353 K coincides with the experimental spectrum of the TEMPO radical in the polymer globule obtained by the “quenching” experiment (Figure 3).

**Figure 5:**
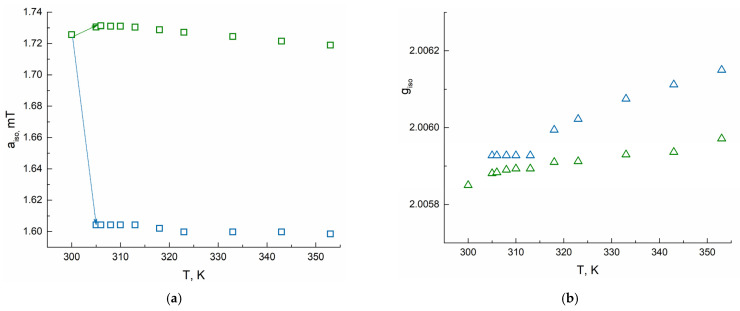
Change of *a*_iso_ (**a**) and *g*_iso_ (**b**) of TEMPO radical in PNIPAM solution with heating according to EPR spectra simulation. Green squares and triangles correspond to ***A*** particles, blue ones correspond to ***B*** particles.

Figure 5 shows changes of *a*_iso_ and *g*_iso_ of radicals ***A*** and ***B*** as a function of temperature. The complete simulation results obtained from EPR spectra fitting at several temperatures are collected in Table A1 of Appendix A. At 305–313K, the *g*_iso_ values corresponding to ***B*** particles are similar to those of probe ***A*** in aqueous solution, but after 313 K, they rise to 2.00615 at 353 K. In contrast, *a*_iso_ values corresponding to ***B*** probes do not change at 305–353 K being equal to 1.60 mT. The average rotation correlation time of particles ***B*** is 1300 ps at 305–318 K. After 318 K, a two-fold decrease of *τ_corr_* to 620 ps resulting in narrowing of the line width of the EPR signal of the ***B*** probes is observed, signaling that TEMPO molecules in the globule become more mobile. The mole fraction of type ***B*** probes rises upon heating to 353 K to 70%.

## 4. Discussion

All the applied methods: DSC, spectrophotometry, and the EPR spin probe technique determine the same value of LCST (305 K) in aqueous PNIPAM solutions of polymers I and II, which is consistent with the literature data [33]. Coil-to-globule transition on PNIPAM aqueous solutions studied by the EPR spin probe method occurs in a narrow temperature range of 8–10 K. Thus, in solutions, hydrophilic bonds between water molecules and PNIPAM individual chains quickly degrade, and the rapid formation of polymer globules takes place. To the contrary, Junk et al. also fixed LCST at 305 K in PNIPAM-based photocrosslinked films swollen in water, but the temperature range of the collapse as measured by spin-probe CW EPR spectroscopy was substantially broadened to 40–50 K [19]. Therefore, only one type of radical (***A*** probes) prevails in the aqueous solution of PNIPAM below LSCT (305 K). Nevertheless, at 302–304 K, some part of TEMPO molecules is captured into polymer globules, transferring to ***B*** probes and testifying to the generation of heterogeneities that begin to collapse at 305 K, when the coil-to-globule transition takes place.

We suppose that the observed increase in g-factor values of ***B*** particles in the globules with increasing temperature occurs due to the breakdown of hydrogen bonds between water molecules and the N–O^●^ group of TEMPO [34,35]. At higher temperatures, these complexes decompose, and *g*_iso_ rises to lower polar values corresponding to individual TEMPO radicals. In addition, water molecules may come out of the globules while heating, making the media surrounding TEMPO radicals more hydrophobic. Such evolution from molten globule to tight globule was previously observed by Wang et al. [36] by laser scattering for PNIPAM with narrow mass distribution, and it may also lead to the change of *g*_iso_ value to be less polar.

The changes of content in *A* probes obtained from spectra simulation are similar to the normalized amplitude changes of the high-field component of the full TEMPO spectrum in PNIPAM solutions (see Figure 2 and Table A1). Therefore, the amplitude changes may be used not only for LCST determination but for estimation of the content of probe molecules ***A***(χ_A_) in the aqueous solution after the globule collapse. Since χ_A_+ χ_B_ = 1, the molar fraction of ***B*** probes could be valued, too. The content of probes in the collapsed globules relates to an affinity of the probe to the polymer and amphiphilicity of the collapsed globules. Thus, the measuring of amplitudes of EPR spectra of probe molecules in thermoresponsive polymers aqueous solutions gives the opportunity to determine and predict its properties using the spin-probe technique without making challenging spectra simulations.

## 5. Conclusions

The CW EPR method with the spin-probe technique and “quenching” approach was applied for studying coil-to-globule phase transition in PNIPAM aqueous solution upon heating at 298–353 K. The broadening of fast-moving radicals in water by spin exchange with Cu(II) ions allows obtaining individual spectra of the spin probe in polymer globule after collapsing and reliable spin-Hamiltonian parameters of both types of TEMPO probes in solutions. In addition, the “quenching” approach has shown the formation of inhomogeneities in PNIPAM aqueous solution at 2–3 degrees below the critical temperature, whereas the temperature range of the collapse in PNIPAM-based photocrosslinked films swollen in water is substantially broadened to 40–50 K. A simple analysis based on amplitude measurement of TEMPO EPR spectra registered at different temperatures gives a cloud point and allows estimating the content of probe in collapsed globule. According to EPR spectra simulations, the formation of dense globules at high temperatures through molten ones at the first step takes place in PNIPAM solutions.

## Data Availability

The data presented in this study are available on request from the corresponding author.

## Appendix A

### EPR Spectra Simulation Details

All CW EPR spectra simulations were performed using homemade scripts for MATLAB program employing the Easyspin (v. 5.2.28) toolbox [23]. Quality of fitting was controlled by the calculation of root-mean-square deviation for the difference spectra. Spectral and dynamic parameters were obtained by fitting simulated EPR spectra to experimental data using least-squares fitting algorithms.

The spectra of TEMPO radical (type ***A***) in solutions were simulated using a model based on fast-motion implemented as a ‘garlic’ function in Easyspin. Line broadening was described by isotropic rotation correlation time, and by additional convolutional line broadening caused mostly by unresolved hyperfine splitting between unpaired electron and nuclear spin (usually ^1^H). As initial values for the diagonal elements of the g-tensor and hyperfine splitting tensor on ^14^N nuclei for type ***A*** probes, the following values were used:

g_x_ = 2.0092, g_y_ = 2.0062, g_z_ = 2.0022;

A_xx_ = 0.71 mT, A_yy_ = 0.70 mT, A_zz_ = 3.71 mT.

In addition to splitting on ^14^N nuclei in the case of type ***A*** probes, isotropic hyperfine splitting on ^13^C isotopes of six CH_3_-groups with *a*_iso_ = 0.54 mT was considered. To simplify the modeling procedure, we change abundances for carbon isotopes instead of adding six paramagnetic nuclei.

Simulations of the spectra of radicals in polymer globule (type ***B***) were made in a slow-motion regime using the ‘chili’ function from the Easyspin program. The slow-motion model ‘chili’ is based on the Schneider–Freed theory [24], solving equations for slow-tumbling nitroxides. Line broadening was described by the anisotropic rotation correlation time and additional Gaussian line broadening. To start fitting, the following diagonal values of g-tensor and hyperfine interaction a-tensor were used:

g_x_ = 2.0098, g_y_ = 2.0062, g_z_ = 2.0022;

A_xx_ = 0.71 mT, A_yy_ = 0.70 mT, A_zz_ = 3.35 mT.

The initial values of magnetic parameters were taken from [37] and corrected using experimental isotropic *g*_iso_ and *a*_iso_ of TEMPO radical in different solvents [30]. During the simulation, only g_x_ and A_zz_ components were varied, being more sensitive to the environment. Isotropic g_iso_ and a_iso_ were calculated as an average value of diagonal elements:

$$g_{\mathrm{iso}}=\frac{1}{3}(g_{x}+g_{y}+g_{z})$$

$$a_{\mathrm{iso}}=\frac{1}{3}(A_{\mathrm{xx}}+A_{\mathrm{yy}}+A_{\mathrm{zz}})$$

The average rotational correlation time *τ_corr_* was calculated using the following equations from diagonal elements of diffusion tensor D_r_ = [*D_xx_ D_yy_ D_zz_*]:

$$D_{r}=\frac{1}{6\tau_{corr}}$$

$$\tau_{\mathrm{corr}}\;=\frac{1}{6\sqrt[{3}]{D_{xxD_{yy}D_{zz}}}}$$

**Table A1:** Selected spectral parameters from simulation TEMPO EPR spectra in 10 wt% aqueous solutions at 300–353 K.

T, K	Type A			Type B				
	*g*_iso_	*a*_iso_, mT	*τ_corr_*, ps	*g*_iso_	*a*_iso_, mT	*τ_corr_*, ns	χ_B_, % (Polymer I)	χ_B_, % (Polymer II)
300	2.00585	1.72	16	-	-	-	0	0
305	2.00588	1.73	10	2.00593	1.60	1.26	26.5	44.6
306	2.00588	1.73	8	2.00593	1.60	1.31	50.0	52.6
308	2.00589	1.73	8	2.00593	1.60	1.31	56.1	54.5
310	2.00589	1.73	5	2.00593	1.60	1.31	58.3	55.9
313	2.00589	1.73	5	2.00593	1.60	1.31	61.7	58.8
318	2.00591	1.73	2	2.00599	1.60	1.31	63.1	62.0
323	2.00591	1.73	2	2.00602	1.60	1.25	64.5	63.2
333	2.00593	1.72	2	2.00608	1.60	1.01	65.9	64.3
343	2.00594	1.72	2	2.00611	1.60	0.86	68.8	67.2
353	2.00597	1.72	2	2.00615	1.60	0.74	70.1	69.9

## References

[1] Heskins M., Guillet J.E. (1968). Solution Properties of Poly(N-isopropylacrylamide). J. Macromol. Sci. Part A-Chem..

[2] Aseyev V., Tenhu H., Winnik F.M. (2011). Non-Ionic Thermoresponsive Polymers in Water. Adv. Polym. Sci..

[3] Pasparakis G., Tsitsilianis C. (2020). LCST Polymers: Thermoresponsive Nanostructured Assemblies towards Bioapplications. Polymer.

[4] Peppas N.A., Hilt J.Z., Khademhosseini A., Langer R. (2006). Hydrogels in Biology and Medicine: From Molecular Principles to Bionanotechnology. Adv. Mater..

[5] Ward M.A., Georgiou T.K. (2011). Thermoresponsive Polymers for Biomedical Applications. Polymers.

[6] Nash M.E., Fan X., Carroll W.M., Gorelov A.V., Barry F.P., Shaw G., Rochev Y.A. (2013). Thermoresponsive Substrates Used for the Expansion of Human Mesenchymal Stem Cells and the Preservation of Immunophenotype. Stem Cell Rev. Rep..

[7] Frolova A., Ksendzov E., Kostjuk S., Efremov Y., Solovieva A., Rochev Y., Timashev P., Kotova S. (2021). Thin Thermoresponsive Polymer Films for Cell Culture: Elucidating an Unexpected Thermal Phase Behavior by Atomic Force Microscopy. Langmuir.

[8] Cao M., Wang Y., Hu X., Gong H., Li R., Cox H., Zhang J., Waigh T.A., Xu H., Lu J.R. (2019). Reversible Thermoresponsive Peptide–PNIPAM Hydrogels for Controlled Drug Delivery. Biomacromolecules.

[9] Doberenz F., Zeng K., Willems C., Zhang K., Groth T. (2020). Thermoresponsive Polymers and Their Biomedical Application in Tissue Engineering—A Review. J. Mater. Chem. B.

[10] Lu H., Leng J., Du S. (2013). A Phenomenological Approach for the Chemo-Responsive Shape Memory Effect in Amorphous Polymers. Soft Matter.

[11] Zhang Q., Weber C., Schubert U.S., Hoogenboom R. (2017). Thermoresponsive Polymers with Lower Critical Solution Temperature: From Fundamental Aspects and Measuring Techniques to Recommended Turbidimetry Conditions. Mater. Horiz..

[12] Ding Y., Ye X., Zhang G. (2005). Microcalorimetric Investigation on Aggregation and Dissolution of Poly(N-Isopropylacrylamide) Chains in Water. Macromolecules.

[13] Antheunis H., van der Meer J.-C., de Geus M., Heise A., Koning C.E. (2010). Autocatalytic Equation Describing the Change in Molecular Weight during Hydrolytic Degradation of Aliphatic Polyesters. Biomacromolecules.

[14] Kurzbach D., Junk M.J.N., Hinderberger D. (2013). Nanoscale Inhomogeneities in Thermoresponsive Polymers. Macromol. Rapid Commun..

[15] Winnik F.M., Ottaviani M.F., Bossmann S.H., Garcia-Garibay M., Turro N.J. (1992). Consolvency of Poly(N-Isopropylacrylamide) in Mixed Water-Methanol Solutions: A Look at Spin-Labeled Polymers. Macromolecules.

[16] Kokorin A. (2012). Nitroxides—Theory, Experiment and Applications.

[17] Drescher M., Jeschke G. (2012). EPR Spectroscopy.

[18] Kurzbach D., Reh M.N., Hinderberger D. (2011). Nanoscale Inhomogeneities in Thermoresponsive Triblock Copolymers. ChemPhysChem.

[19] Junk M.J.N., Jonas U., Hinderberger D. (2008). EPR Spectroscopy Reveals Nanoinhomogeneities in the Structure and Reactivity of Thermoresponsive Hydrogels. Small.

[20] Rochev Y., O’Halloran D., Gorelova T., Gilcreest V., Selezneva I., Gavrilyuk B., Gorelov A. (2004). Rationalising the Design of Polymeric Thermoresponsive Biomaterials. J. Mater. Sci. Mater. Med..

[21] Caragheorgheopol A., Schlick S. (1998). Hydration in the Various Phases of the Triblock Copolymers EO13PO30EO13 (Pluronic L64) and EO6PO34EO6 (Pluronic L62), Based on Electron Spin Resonance Spectra of Cationic Spin Probes. Macromolecules.

[22] Eaton G.R., Eaton S.S., Barr D.P., Weber R.T. (2010). Quantitative EPR.

[23] Stoll S., Schweiger A. (2006). EasySpin, a Comprehensive Software Package for Spectral Simulation and Analysis in EPR. J. Magn. Reson..

[24] Schneider D.J., Freed J.H., Berliner L.J., Reuben J. (1989). Calculating Slow Motional Magnetic Resonance Spectra. Spin Labeling: Theory and Applications.

[25] Yanase K., Buchner R., Sato T. (2020). Microscopic Insights into the Phase Transition of Poly(N-Isopropylacrylamide) in Aqueous Media: Effects of Molecular Weight and Polymer Concentration. J. Mol. Liq..

[26] Hunold J., Eisermann J., Brehm M., Hinderberger D. (2020). Characterization of Aqueous Lower-Polarity Solvation Shells Around Amphiphilic 2,2,6,6-Tetramethylpiperidine-1-Oxyl Radicals in Water. J. Phys. Chem. B.

[27] Salikhov K.M. (2019). Current State of the Spin Exchange Theory in Dilute Solutions of Paramagnetic Particles. New Paradigm of Spin Exchange and Its Manifestations in EPR Spectroscopy. Physics-Uspekhi.

[28] Salikhov K.M. (2020). New Paradigm of Spin Exchange and Its Manifestations in EPR Spectroscopy. Appl. Magn. Reson..

[29] Salikhov K.M. (2016). Consistent Paradigm of the Spectra Decomposition into Independent Resonance Lines. Appl. Magn. Reson..

[30] Kecki Z., Lyczkowski Z. (1986). KW Critical Comparison of Empirical Systems Used to Describe Solvent Properties. J. Solution Chem..

[31] Ellison W.J. (2007). Permittivity of Pure Water, at Standard Atmospheric Pressure, over the Frequency Range 0–25THz and the Temperature Range 0–100 °C. J. Phys. Chem. Ref. Data.

[32] Korson L., Drost-Hansen W., Millero F.J. (1969). Viscosity of Water at Various Temperatures. J. Phys. Chem..

[33] Halperin A., Kröger M., Winnik F.M. (2015). Poly( N-isopropylacrylamide) Phase Diagrams: Fifty Years of Research. Angew. Chemie Int. Ed..

[34] Franchi P., Lucarini M., Pedrielli P., Pedulli G.F. (2002). Nitroxide Radicals as Hydrogen Bonding Acceptors. An Infrared and EPR Study. ChemPhysChem.

[35] Rinkevicius Z., Murugan N.A., Kongsted J., Aidas K., Steindal A.H., Ågren H. (2011). Density Functional Theory/Molecular Mechanics Approach for Electronic g-Tensors of Solvated Molecules. J. Phys. Chem. B.

[36] Wu C., Wang X. (1998). Globule-to-Coil Transition of a Single Homopolymer Chain in Solution. Phys. Rev. Lett..

[37] Lebedev Y.S., Grinberg O.Y., Dubinsky A.A., Poluektov O.G. (1992). Investigation of Spin Labels and Probes by Millimeter Band EPR. Bioactive Spin Labels.

